# Exosomes orchestrate immune homeostasis in acute ischemia-reperfusion flap injury and chronic wounds: the TLR4/NF-κB-STAT3 R-ratio balance decision model, engineering optimization and translational clinical strategy

**DOI:** 10.3389/fimmu.2026.1930726

**Published:** 2026-08-25

**Authors:** Qin Yue, Jinhao Chen, Lin Liu, Hui Liu, Xinyi Zeng

**Affiliations:** Yangtze University Health Science Center, Jingzhou, Hubei, China

**Keywords:** chronic wounds, exosomes, flap ischemia-reperfusion injury, immune homeostasis, immune microenvironment, TLR4/NF-κB-STAT3

## Abstract

Flap ischemia-reperfusion injury (IRI) and chronic refractory wounds are intractable clinical challenges in the field of wound repair. Both pathological conditions share a core pathological hallmark: disrupted homeostasis of the local wound inflammatory immune microenvironment, yet they exhibit distinct patterns of inflammatory dysregulation. Mesenchymal stem cell-derived exosomes exert bidirectional immunomodulatory effects. They inhibit the TLR4/NF-κB signaling pathway to alleviate the acute inflammatory storm triggered by IRI, whereas they activate the STAT3 pathway to reinitiate tissue repair in chronic wounds. Nevertheless, a unified theoretical framework explaining the environment-dependent functional switch of exosomes remains absent in current research, hindering the development and clinical translation of targeted engineered exosomes. To fill this research gap, we perform a systematic review integrating five pivotal inflammatory signaling pathways, including TLR4/NF-κB, STAT3, TGF-β/Smad, NLRP3 and MAPK. We establish a TLR4/NF-κB-STAT3 signal ratio balance decision model to uniformly interpret inconsistent findings from existing *in vitro* and *in vivo* experiments. This model verifies that the concentrations of inflammatory cytokines, oxidative stress levels, and immune cell subsets within the wound microenvironment coordinately regulate the activation intensity ratio (R value) of the TLR4/NF-κB and STAT3 pathways, which ultimately determines whether exosomes exert anti-inflammatory or pro-repair effects. Unlike conventional strategies that only enhance a single therapeutic function, the proposed model provides quantitative evidence for the targeted design of microenvironment-adaptive intelligent exosomes. This review further summarizes standardized quality control protocols and stratified clinical trial designs for exosomes, objectively discusses the limitations of current translational research, and puts forward multiple verifiable follow-up experimental hypotheses. Collectively, this work constructs a comprehensive theoretical framework for precise immune regenerative therapies targeting flap IRI and chronic wounds.

## 1 Introduction

Flap transplantation is a core surgical technique in plastic surgery, burn repair and trauma reconstruction, widely applied to repair large-area soft tissue defects and complex tissue injuries after tumor resection. Nevertheless, postoperative flap ischemia-reperfusion injury (IRI) represents the most prevalent and severe clinical complication, which directly reduces flap survival rates, raises patients’ risk of disability and increases overall medical burdens (1). Excessive immune-inflammatory activation triggered by IRI serves as the central driver of flap necrosis (2): rapid and severe inflammatory responses erupt at wound sites following blood reperfusion, accompanied by massive infiltration of neutrophils and M1-type macrophages (3). These immune cells secrete excessive pro-inflammatory cytokines, reactive oxygen species (ROS) and chemokines, disrupt vascular endothelial integrity, exacerbate microcirculation disorders, and ultimately induce flap edema, hemorrhage and progressive tissue necrosis (4). Unlike fulminant inflammatory damage in acute IRI, chronic wounds including diabetic foot ulcers, pressure ulcers, venous ulcers and residual burn wounds exhibit distinct immune imbalance, trapped in a vicious cycle of persistent inflammation and impaired tissue repair (5–7). Acute wound healing follows a sequential “inflammation-proliferation-remodeling” cascade, while chronic wounds are persistently locked in an M1 macrophage-dominated hyperinflammatory state (8, 9). Sustained aberrant activation of the TLR4/NF-κB pathway leads to constant overexpression of pro-inflammatory cytokines, delayed clearance of inflammatory cells and tissue debris (10), and failure to shift from pro-inflammatory to repair-permissive immune phenotypes (11). Collectively, both acute flap IRI-induced necrosis and non-healing chronic wounds stem from suppressed tissue regeneration driven by dysregulated inflammatory immune microenvironments.

As pivotal mediators of intercellular signal communication, exosomes exert indispensable roles in regulating inflammation and immune homeostasis (12). These bilayered membrane vesicles ranging from 30 to 150 nm encapsulate functional proteins, nucleic acids and lipids derived from parent cells. Efficiently internalized by target cells, exosomes precisely modulate cellular phenotypes and biological functions (13), and broadly participate in innate and adaptive immune regulation during sterile inflammation, infectious inflammation and autoimmune disorders (14). Mesenchymal stem cells (MSCs, particularly adipose-, bone marrow- and umbilical cord-derived MSCs) constitute the predominant source of therapeutic exosomes at present (15). MSC-derived exosomes modulate the activation and polarization of immune cells via multiple pathways while retaining low immunogenicity (16). Cumulative evidence confirms that exosomes regulate core inflammatory signaling cascades such as TLR4/NF-κB (17) and STAT3 (18). In acute IRI models, they primarily restrain excessive TLR4/NF-κB activation, curb inflammatory storms and protect vascular endothelium; in contrast, they tend to activate the STAT3 pathway within chronic wound microenvironments to mitigate persistent inflammation and initiate tissue repair programs. Therefore, exosomes are regarded as promising agents with bidirectional immunomodulatory capacities and have become a cutting-edge research hotspot for precise wound treatment.

However, a critical long-standing contradiction remains unaddressed in existing research: why do identical or homologous exosomes exert opposing pathway preferences and functional phenotypes in acute IRI versus chronic wounds? Current publications merely describe pathway alterations in individual disease models and attribute divergent effects to microenvironmental differences in a simplistic manner, without establishing an integrated theoretical framework to decode the decision-making mechanism by which exosomes sense microenvironmental cues and switch functional modes. The absence of such a mechanistic model confines the engineering modification of exosomes to empirical strategies that simply overexpress bioactive cargos to statically amplify anti-inflammatory or regenerative effects, hindering the development of truly microenvironment-adaptive precise immune interventions.

To resolve the above research bottlenecks, this study originally proposes the TLR4/NF-κB-STAT3 signal balance decision model. This model posits that the anti-inflammatory or pro-repair functions of exosomes are not inherent properties, but dynamic outcomes determined by the signal intensity ratio of TLR4/NF-κB to STAT3, which is jointly modulated by local microenvironmental inflammatory cytokine levels, oxidative stress magnitude and immune cell subset proportions. When the signal ratio exceeds a specific threshold, exosomes predominantly mediate the resolution of acute inflammation (acting as an “anti-inflammatory brake”); when the ratio falls below this threshold, exosomes switch to alleviating chronic inflammation and facilitating tissue regeneration (pro-repair phenotype). This review systematically integrates five core pathways (TLR4/NF-κB, STAT3, TGF-β/Smad, NLRP3, MAPK) and defines the distinct functional roles of each pathway within the overall regulatory network, reinterpreting seemingly contradictory experimental observations reported in previous literature. Based on this balance model, we further propose an innovative design principle for intelligent exosomes, breaking the conventional strategy of single-function enhancement, and constructing an adaptive regulatory system capable of sensing microenvironmental signals and dynamically balancing inflammatory and regenerative signaling. Finally, this review systematically summarizes feasible translational routes and core bottlenecks restricting clinical application of exosomes, and puts forward three verifiable scientific hypotheses, providing a novel and unified theoretical system for precise immunotherapy of wounds.

Three prominent limitations persist in current research on exosome-mediated wound repair. First, existing studies separately report the single functional phenotype of exosomes in either acute or chronic wounds, lacking a unified decision-making mechanism to explain bidirectional functional switching and reconcile conflicting experimental data across publications. Second, previous reviews merely enumerate the biological functions of individual inflammatory pathways, without constructing a network-based quantitative regulatory system for coordinated multi-pathway crosstalk. Third, engineered exosome modification still relies on static functional enhancement, lacking theoretical support for intelligent design guided by microenvironmental signal ratios. Accordingly, this review centers on the core scientific question: how do homologous exosomes switch their functions in response to microenvironmental differences between acute hyperinflammation and chronic low-grade inflammation, and whether this signal ratio mechanism can guide the precise design of intelligent exosomes and individualized clinical therapies?

To address this question, this work innovatively establishes a quantitative balance model defined as R = TLR4/NF-κB signal intensity/STAT3 signal intensity, which uniformly elucidates the divergent immunomodulatory patterns of exosomes in acute and chronic wounds. By delineating the functional division of the five core pathways within the R-value regulatory network, we construct a complete regulatory system for wound immune balance. Furthermore, this study establishes novel modification criteria and translational strategies for microenvironment-responsive intelligent exosomes based on the proposed model, filling the theoretical gap spanning mechanistic interpretation, engineered modification and clinical translation.

## 2 Biogenesis of exosomes

Exosomes are bilayer lipid membrane-enclosed extracellular vesicles that undergo intracellular assembly and extracellular secretion through the canonical endosomal pathway. Their biogenesis initiates with inward invagination of the plasma membrane to generate early endosomes. Cells absorb extracellular substances and internalize membrane proteins via early endosomes, which further differentiate and mature into late endosomes, also termed multivesicular bodies (MVBs). Intraluminal vesicles (ILVs) are produced by inward budding of the MVB membrane, a process co-regulated by the endosomal sorting complex required for transport (ESCRT)-dependent and ESCRT-independent pathways (19).

**Figure 1:**
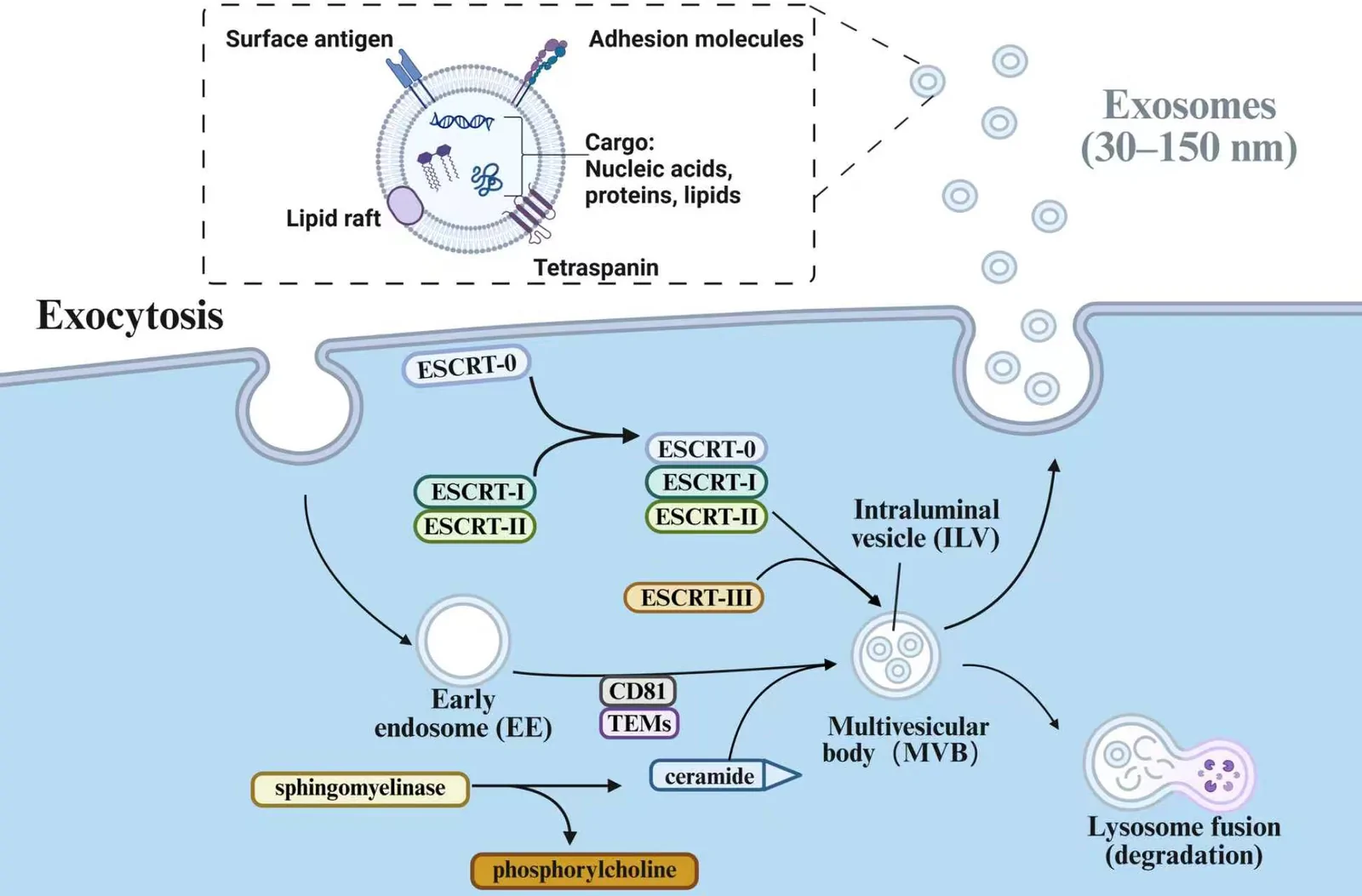
Schematic diagram of exosome biogenesis. Exosomes are formed through the canonical endosomal pathway. An early endosome matures into an MVB, where an ILV is generated via ESCRT-dependent or ceramide- and tetraspanin-mediated ESCRT-independent pathways. Finally, the MVB fuses with the plasma membrane to release exosomes, or fuses with lysosomes for cargo degradation. Created with BioRender.com. MVB, multivesicular body; ILV, intraluminal vesicle; ESCRT, endosomal sorting complex required for transport.

The ESCRT machinery consists of four subunits: ESCRT-0, ESCRT-I, ESCRT-II and ESCRT-III, each with distinct roles in facilitating ILV biogenesis. ESCRT-0 specifically recognizes ubiquitinated substrate proteins via its ubiquitin-binding domain (UBD) to initiate cargo sorting. ESCRT-I and ESCRT-II trigger membrane curvature and construct the budding architecture of endosomes, whereas ESCRT-III mediates vesicle scission to sequester sorted cargos and ultimately produce ILVs (20). The ESCRT system is not the sole regulator of ILV formation and cargo sorting; multiple ESCRT-independent pathways also participate in exosome biogenesis (21). Sphingomyelinase hydrolyzes sphingomyelin to generate ceramide, which promotes the assembly of lipid rafts and drives membrane invagination and budding to facilitate ILV formation (22). Meanwhile, tetraspanin-enriched microdomains (TEMs) formed with the mediation of CD81 not only transmit intracellular signaling cascades but also provide vital membrane platforms for sorting and aggregating exosomal proteins (23). Subsequently, a subset of MVBs fuse with lysosomes to degrade internal ILVs and their cargos, while the remaining MVBs fuse with the plasma membrane and release ILVs into the extracellular space to form mature exosomes (**Figure 1**).

The assembly and sorting machinery of exosomes directly shapes their molecular cargo profiles, acting as the fundamental prerequisite for exosomes to exert divergent immunomodulatory effects. Notably, exosomal cargo sorting is not a static, fixed procedure. Microenvironmental cues including inflammatory cytokines, hypoxia and oxidative stress can dynamically reshape the cargo packaging preference of parent cells (24). For instance, upon TNF-α stimulation or hypoxic treatment, cells selectively enrich inflammation-associated miRNAs (such as miR-146a and miR-155) and immune-related proteins, thereby remodeling the immune signaling repertoire carried by exosomes (25, 26). Independent studies have validated that the microenvironments of acute IRI and chronic wounds can remodel exosomal cargos respectively (27). Nevertheless, few investigations have integrated the molecular discrepancies between these two wound phenotypes into a unified functional regulatory framework, which constitutes the core scientific issue addressed by the TLR4/NF-κB-STAT3 balance decision model constructed in the subsequent sections of this review.

## 3 Immune dysregulation in flap IRI and chronic wounds

### 3.1 Flap IRI: tissue necrosis driven by acute inflammatory storms

Under the hyperinflammatory microenvironment induced by flap IRI, hypoxia-reoxygenation stress in local tissues trigger an acute pathological injury cascade. Damaged cells secrete abundant damage-associated molecular patterns (DAMPs), which elicit robust activation of innate immunity (28). These signaling molecules continuously activate inflammatory cascades and stimulate massive secretion of pro-inflammatory mediators including TNF-α and IL-6, forming severe aseptic acute inflammatory storms (29, 30). Massive neutrophil infiltration accompanied by the release of neutrophil extracellular traps (NETs) disrupts endothelial barrier and induces microthrombosis within microcirculation, further aggravating the vicious cycle of regional ischemia and hypoxia (31, 32). Meanwhile, these signals persistently drive macrophages toward the pro-inflammatory M1 phenotype, establishing a microenvironment rich in pro-inflammatory factors. This amplifies oxidative stress and pyroptosis in resident tissue cells, ultimately accelerating flap edema and necrosis and drastically reducing postoperative flap survival rates (33).

### 3.2 Chronic wounds: impaired inflammation resolution and stalled tissue repair

In stark contrast, chronic wounds such as diabetic foot ulcers and venous ulcers are defined by persistent inflammation stasis rather than acute inflammatory bursts (34). The core pathological feature lies in chronic sustained overactivation of the TLR4/NF-κB pathway, which permanently blocks inflammation resolution. Macrophage polarization homeostasis collapses: pro-inflammatory M1 macrophages accumulate persistently while M2 reparative polarization is suppressed, preventing physiological attenuation and clearance of chronic inflammatory signals (35). The innate immune system exhibits low-grade hyperactivation, while adaptive immunity becomes compromised, manifested by T cell senescence and disrupted antigenic responses that abolish normal regenerative cascades (36). Additionally, tissues synthesize insufficient pro-resolving mediators, leaving pro-inflammatory signaling pathways constitutively dominant. This persistently inhibits fibroblast activation, angiogenesis and ordered extracellular matrix (ECM) remodeling, leading to an intractable pathological stalemate characterized by unresolved inflammation and halted tissue regeneration (37).

The above comparison highlights a core distinction: flap IRI features fulminant aseptic inflammatory storms and pan-pathway overactivation. Within this hyperinflammatory microenvironment, exosomes act as potent “inflammatory brakes”. By modulating both innate and adaptive immunity, exosomes construct an immune protective niche during acute injury and stabilizes vascular endothelial barriers, thereby laying a cellular foundation for improved flap survival. In contrast, chronic wounds are plagued by sustained pathway activation, defective inflammation resolution and long-term suppression of regenerative processes. Herein, exosomes exert dual modulatory effects: they suppress persistent pro-inflammatory signaling, rectify skewed immune responses, and reinitiate inflammation resolution and tissue regeneration cascades to fundamentally resolve the immunopathological defects of chronic wounds. Nevertheless, a fundamental contradiction remains unaddressed: why can identical exosomes exert opposing regulatory effects on signaling pathways contingent on distinct microenvironment contexts? Existing studies remain merely descriptive and fail to uncover the intrinsic decision-making mechanism governing this functional shift. In fact, the functional bias of exosomes is not fixed but dynamically dictated by the signal intensity ratio shaped by the surrounding inflammatory microenvironment.

## 4 TLR4/NF-κB-STAT3 signal balance decision model for exosomes-mediated wound immune regulation

Exosomes exert bidirectional regulatory effects, as they suppress TLR4/NF-κB cascades and activate STAT3 signaling in flap IRI and chronic wounds, respectively. Nevertheless, current studies fail to decipher the intrinsic logic governing this functional switch. To address this critical research gap, this review establishes the “TLR4/NF-κB-STAT3 signal balance decision model” and systematically dissects the distinct biological roles of five core signaling pathways within this framework.

### 4.1 Mathematical definition and fundamental assumptions of the model

The core variable of the model is defined as the ratio between TLR4/NF-κB signaling intensity and STAT3 signaling intensity within the local microenvironment, expressed as:


$$R=\frac{Signal\left(TLR4/NF-\kappa B\right)}{Signal\left(STAT3\right)}$$

**Figure 2:**
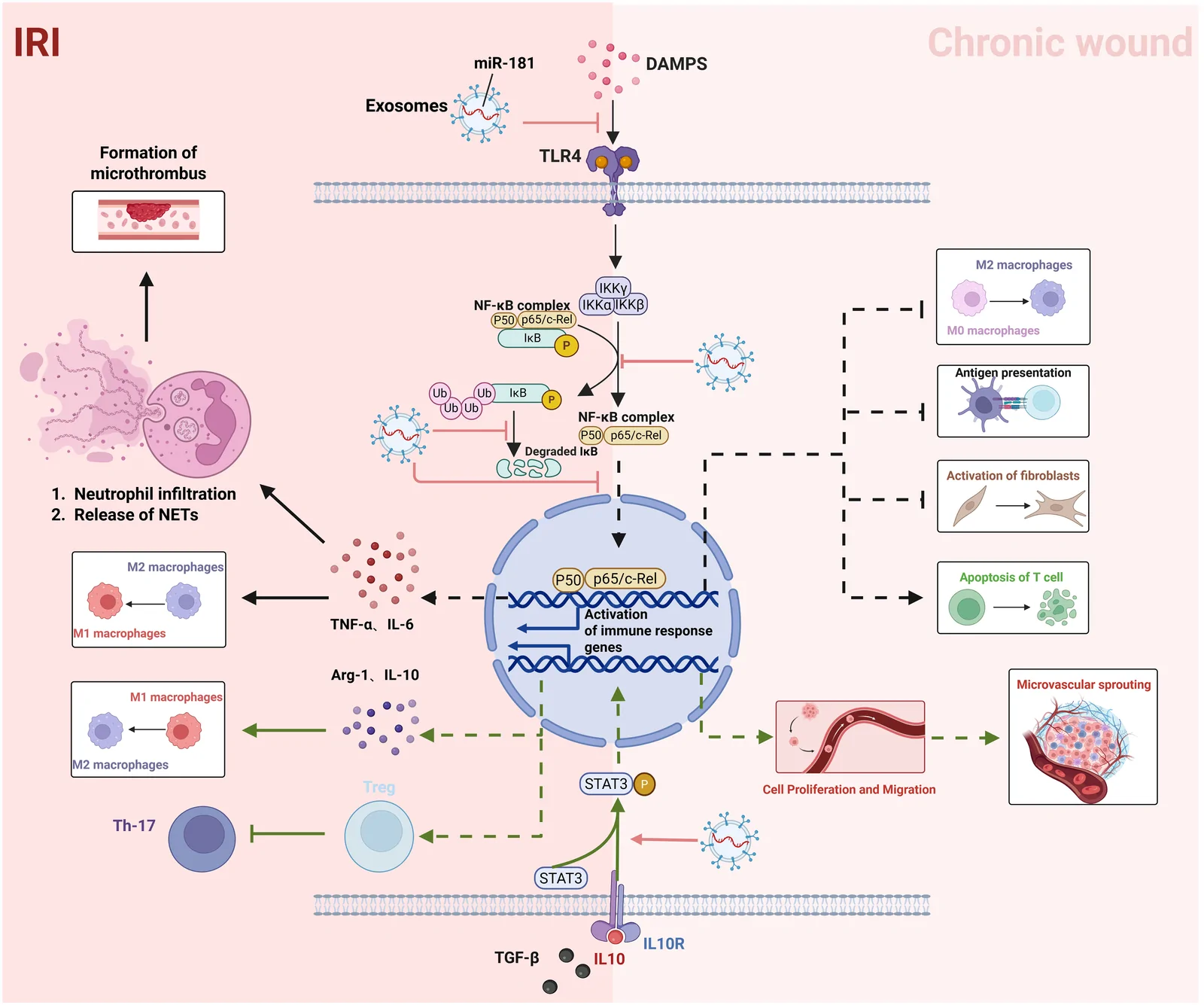
Mechanism diagram of exosomes modulating inflammatory and repairative processes of IRI and chronic wounds. Exosomes suppress the pro-inflammatory TLR4/NF-κB pathway to reduce neutrophil infiltration, M1 macrophage polarization and secretion of pro-inflammatory cytokines. Meanwhile, exosomes activate STAT3 signaling to facilitate M2 macrophage polarization, Treg/Th17 balance, cell proliferation and microvascular sprouting, thereby coordinately remodeling the immune microenvironment of chronic wounds. Created with BioRender.com. IRI, ischemia-reperfusion injury; TLR4, toll-like receptor 4; NF-κB, nuclear factor-κB; STAT3, signal transducer and activator of transcription 3; Treg, regulatory T cell; Th17, T helper 17 cell.

When R exceeds a defined threshold *R_th_*, exosomes initiate an “anti-inflammatory brake” program to predominnantly restrain hyperactive inflammatory storms. When R falls below *R_th_*, exosomes switch to a pro-regenerative program that drives tissue repair. Notably. *R_th_* is not a fixed constant; it is dynamically modulated by microenvironmental cues including reactive oxygen species (ROS) concentration, hypoxia severity, and the relative abundance of immune cell subsets (**Figure 2**). The core hypothesis of this model posits that exosomes do not possess inherent fixed functional phenotypes dictated by their internal cargos. Instead, their functional bias is dynamically governed by microenvironmental signals that tune the relative activation magnitudes of the TLR4/NF-κB and STAT3 pathways.

### 4.2 TLR4/NF-κB signaling pathway: the accelerator of acute inflammation and one core axis of the model

Toll-like receptors (TLRs) belong to pattern recognition receptors (PRRs) and occupy central regulatory positions in innate immune responses (38). Based on subcellular localization, TLRs are categorized into two subgrounds: cell-surface TLR1, TLR2, TLR4, TLR5, TLR6 and TLR10, which detect bacterial and fungal ligands; and intracellular TLR3, TLR7, TLR8 and TLR9, which recognize nucleic acid moieties (39). NF-κB represents the canonical pro-inflammatory signaling cascade. Damage-associated molecular patterns (DAMPs) generated during tissue injury are recognized by the myeloid differentiation factor 2 (MD-2) co-receptor and subsequently presented to TLR4, triggering homodimerization of two TLR4 monomers. Receptor dimerization remodels the conformation of intracellular TIR domains, facilitating recruitment of the TIR-containing adaptor protein myeloid differentiation primary response gene 88 (MyD88). Via its death domain, MyD88 sequentially recruits and activates interleukin-1 receptor-associated kinase 4 (IRAK4) and IRAK1. Activated IRAK1 dissociates from the receptor complex and binds TNF receptor-associated factor 6 (TRAF6), stimulating TRAF6’s ubiquitin ligase activity to catalyze K63-linked ubiquitination of itself and downstream effector proteins (40). These non-degradative ubiquitin chains act as molecular scaffolds to recruit and activate the TGF-β activated kinase 1 (TAK1) complex. Activated TAK1 further confers catalytic activity upon inhibitor of κB kinase (IKK). Functional IKK specifically phosphorylates two conserved serine residues at the N-terminus of NF-κB inhibitor α (IκBα). Phosphorylated IκBα undergoes ubiquitination and subsequent proteasomal degradation. The removal of IκBα unmasks nuclear localization signals on the p65/p50 heterodimer, which rapidly translocates into the nucleus to drive transcription of abundant pro-inflammatory mediators such as TNF-α and IL-1β, ultimately constructing a local pro-inflammatory microenvironment (41).

During the acute phase of flap IRI, massive DAMP release drastically amplifies TLR4/NF-κB signaling, driving R values far above the threshold. By delivering bioative cargos including miR-181 and anti-inflammatory proteins, exosomes target and silence upstream TLR4 adaptor proteins to reduce receptor sensitivity (42). This intervention suppresses IκBα phosphorylation and degradation, blocks nuclear translocation of NF-κB, and downregulates transcription of downstream pro-inflammatory genes, collectively executing the acute inflammatory braking effect (43, 44). In chronic wounds, TLR4/NF-κB exhibits persistent low-grade activation. Although the resulting R value is lower than that observed in acute IRI, it remains elevated enough to sustain pathological inflammation. Exosomes attenuate chronic overactivation of this cascade to abort sustained pro-inflammatory signaling, abolish persistent M1 macrophage polarization (45), and create permissive conditions for inflammation resolution (46). Collectively, the TLR4/NF-κB pathway acts as an inflammatory accelerator within the proposed model, and its signaling magnitude serves as the primary molecular determinant of the R ratio.

### 4.3 STAT3 signaling pathway: the steering wheel of reparative immunity and the other core axis of the model

STATs are nuclear transcription factors that responds to extracellular cytokines and growth factor stimulation, characterized by conserved. Src homology 2 (SH2) and SH3 domains. The STAT protein family encompasses STAT1, STAT2, STAT3, STAT4, STAT5a, STAT5b and STAT6. The STAT3 cascade serves as a rapid signaling route transmitting cytokine signals from membrane receptors into the nucleus (47). Upon binding to the IL-6 receptor α subunit, ligands such as IL-6 recruit the glycoprotein 130 (gp130) signaling subunit and trigger gp130 humodimerization. Although gp130 lacks intrinsic kinase activity, the intracellular Box1 and Box2 motifs recruit JAK1 and JAK2. The close proximity of recruited JAK molecules drives their trans-phosphorylation and activation. Activated JAKs phosphorylate conserved tyrosine residues at the cytoplasmic distal region of gp130; these phosphorylated tyrosine residues form specific docking platforms for the SH2 domains of STAT3 monomers (48). Cytosolic inactive STAT3 monomers bind to receptor phosphorylated tyrosine sites via their SH2 domains, followed by JAK-mediated tyrosine phosphorylation of STAT3 itself. This phosphorylation induces STAT3 conformational rearrangement and dissociation from the receptor, enabling STAT3 homodimer formation and nuclear translocation. Nuclear STAT3 dimers bind to interferon-gamma-activated sequence (GAS) motifs within target genes promoters to initiate transcription of anti-apoptotic mediators including IL-10.

During flap IRI, R values remain markedly elevated, yet exosomes deliver bioactive cargos including IL-10, TGF-β and STAT3-targeted agonists to trigger moderate STAT3 activation. Functional STAT3 signaling facilitates the phenotypic shift of macrophages from pro-inflammatory M1 to reparative M2 subsets (49–51), stabilizes regulatory T cell (Treg) function, suppresses aberrent Th17 activation, and remodels a locally immune-tolerant microenvironment (52). Meanwhile, exosomes mitigate oxidative stress and pyroptosis, reinforce vascular endothelial barriers, and attenuate secondary tissue injury induced by IRI, thereby conferring immune protection for flap survival (53). In chronic wounds, sustained moderate STAT3 activation constitutes the core mechanism underlying exosome-mediated pro-regenerative effects (54). Activated STAT3 acts on fibroblasts, endothelial cells and epidermal stem cells to upregulate genes associated with cell proliferation and migration. It also cooperates with angiogenic signaling to facilitate microvessel sprouting and microvascular remodeling (55, 56). Furthermore, STAT3 drives ordered extracellular matrix (ECM) deposition, accelerates granulation tissue formation and epidermal re-epithelialization (57), alleviates multi-faceted barriers to stalled cutaneous regeneration, and ultimately promotes chronic wounds closure.

Notably, STAT3 signaling magnitude forms the denominator of the R ratio in the proposed model. Sufficient STAT3 activation reduces the R value and shifts exosome bioactivity toward pro-regenerative phenotypes. Accordingly, exosomal modulation of TLR4/NF-κB and STAT3 cascades is not two independent processes; instead, exosomes achieve microenvironment-dependent bidirectional functional switching by tuning the magnitude of the R ratio (**Figure 2**). Under acute hyperinflammatory conditions with extremely high R values, exosomes preferentially suppress TLR4/NF-κB signaling to reduce the numerator of R while mildly enhancing STAT3 activity to raise the denominator, rapidly driving R below the critical threshold *R_th_* (58). For chronic wounds featuring moderate-high basal R levels, exosomes sustain long-term STAT3 activation alongside persistent TLR4/NF-κB suppression, stabilizing R at low levels to maintain reparative immune homeostasis (59).

### 4.4 TGF-β/Smad signaling pathway: the downstream effector of tissue repair in the model

**Figure 3:**
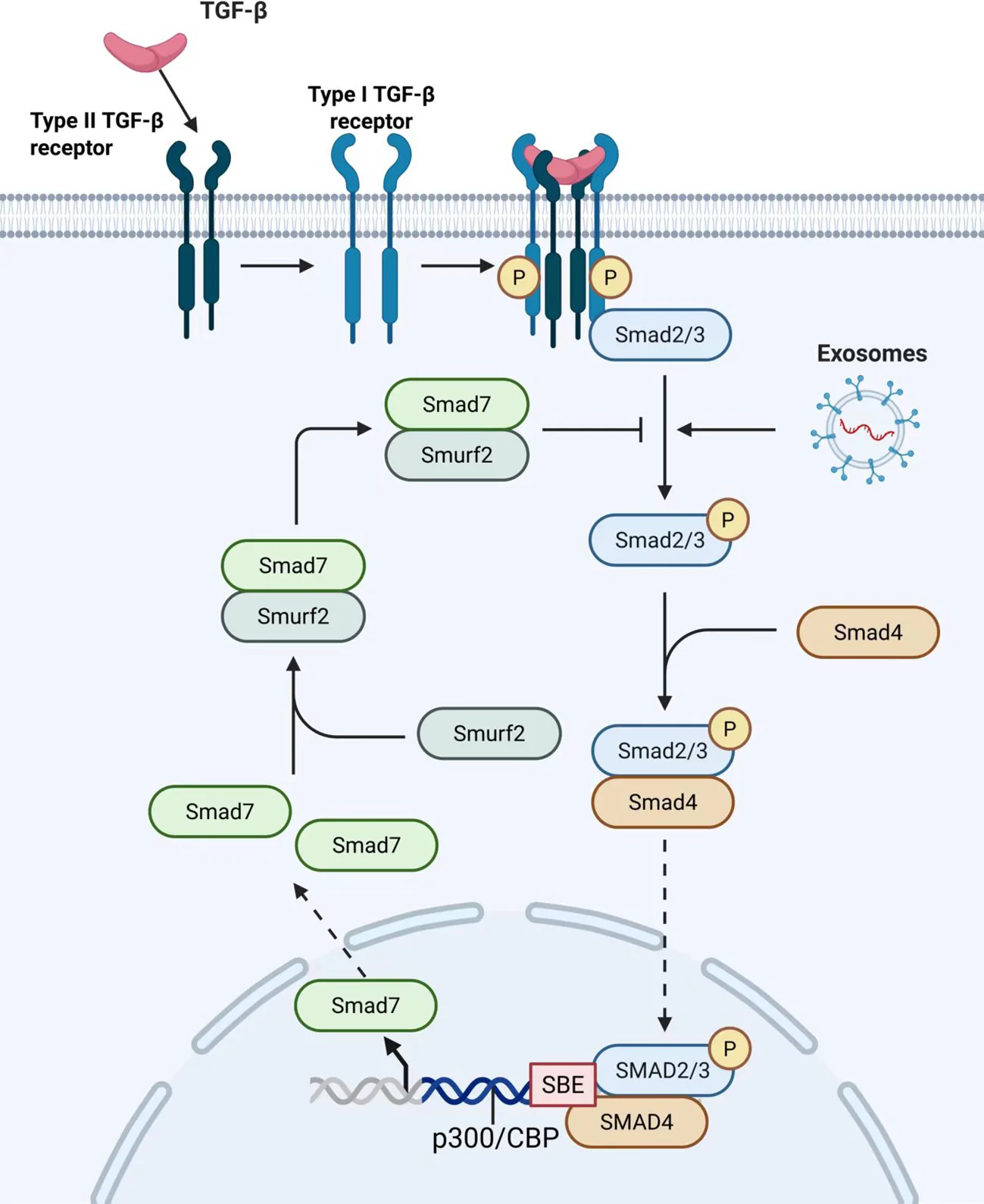
Mechanism diagram of exosomes regulating the TGF-β/Smad signaling pathway. TGF-β binds to the receptor to trigger signal transduction. Phosphorylated Smad2/3 interacts with Smad4, and the complex translocate into the nucleus to modulate the transcription of target gene, while Smad7/Smurf2 exert negatively feedback inhibition on this pathway. Exosomes remodel the ECM and immune microenvironment by facilitating Smad2/3 phosphorylation. Created with BioRender.com. TGF-β, transforming growth factor-β; ECM, extracellular matrix; Smurf2, SMAD specific E3 ubiquitin protein ligase 2.

The TGF-β superfamily comprises multifunctional cytokines that regulate cell growth and differentiation. At least four mammalian isoforms have been identified: TGF-β1, TGF-β2, TGF-β3 and TGF-β1β2 (60). Smad proteins serve as core signal transducers and inducible transcription factors downstream of the TGF-β superfamily. They are functionally categorized into three subgroups: receptor-regulated Smads (R-Smads, Smad1/2/3/5/8), common-partner Smad (Co-Smad: Smad4), and inhibitory Smads (I-Smads: Smad6/7) (61). TGF-β signal transduction relies on Smad-dependent cascades mediated by serine/threonine kinases receptors. Active TGF-β dimers first bind to type II TGF-β receptors, which subsequently recruit and phosphorylate the glycine-serine-rich (GS) domain of the type I receptors to activate their kinase activity. Activated type I receptors specifically target and phosphorylate serine residues within the SSXS motif at the C-terminus of Smad2/3. Phosphorylated Smad2/3 forms heterotrimeric complexes with Smad4 and translocates into the nucleus. Nuclear Smad complexes bind to Smad-binding elements (SBEs) on target gene promoters and recruit transcriptional coactivators such as p300/CBP, thereby initiating the expression of pro-fibrotic and regenerative genes, including type I collagen and α-smooth muscle actin (62). This pathway possesses an intrinsic negative feedback loop. Post-translationally expressed Smad7 translocates to the cytoplasm and recruits the E3 ubiquitin ligase Smurf2 via its PPXY motif. The Smad7-Smurf2 complex binds to activated type I receptors and catalyzes K48-linked ubiquitination, targeting receptors for proteasomal degradation (63) (**Figure 3**). In burn wounds, the strength of this negative feedback directly governs myofibroblast apoptosis. Deficient Smad7 function leads to dysregulated TGF-β signaling and subsequent pathological scar hyperplasia (64).

In the proposed model, the TGF-β/Smad cascade acts as a downstream functional effector whose activity is indirectly modulated by the R value. When exosomes reduce the R value and switch to a pro-regenerative program, TGF-β/Smad signaling is synergistically potentiated. Exosomes enrich TGF-β ligands, enhance Smad2/3 phosphorylation, and suppress Smad7-mediated negative feedback, thereby amplifying TGF-β/Smad signaling activity (65). This cascade consequently facilitates collagen deposition, fibroblast proliferation, and M2 macrophage polarization (66). In flap IRI, moderate TGF-β/Smad activation stabilizes extracellular matrix architecture and alleviates tissue injury (67). In chronic wounds, sustained pathway activation drives the transition from persistent inflammation to proliferative remodeling, serving as a core pro-regenerative force for wound closure (68).

### 4.5 NLRP3 inflammasome: the signal amplifier and pyroptosis switch of the model

**Figure 4:**
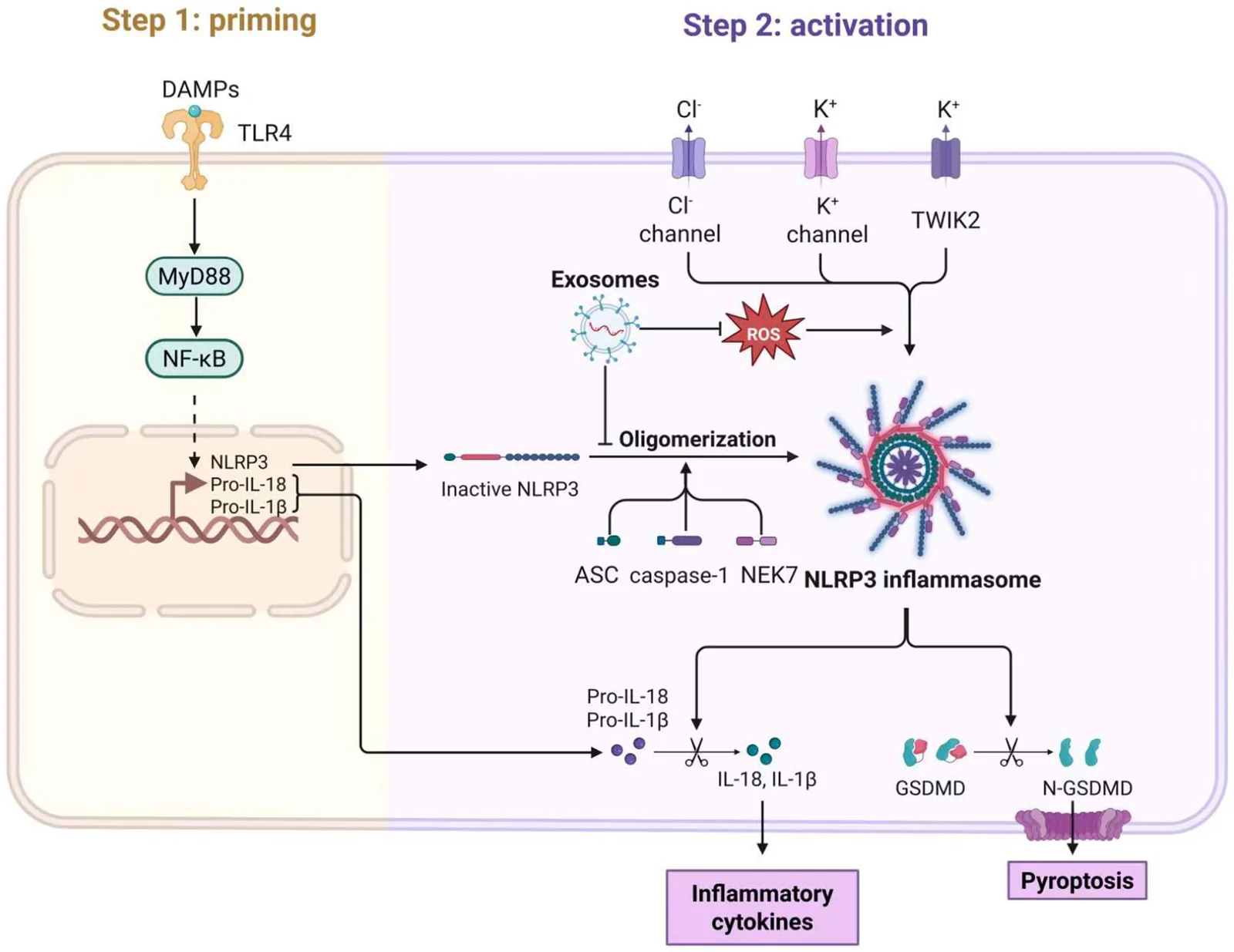
Mechanism diagram of exosomes regulating the NLRP3 inflammasome signaling pathway. This pathway consists of two sequential stages: priming and activation. In the priming stage, the TLR4/NF-κB cascade upregulates the expression of NLRP3 and pro-inflammatory cytokine precursors. In the activation phase, multiple stimuli drive NLRP3 inflammasome assembly, activate caspase-1, cleave downstream substrates, promote the secretion of inflammatory cytokines and ultimately induce pyroptosis. Exosomes alleviate inflammation responses by suppressing NLRP3 inflammasome assembly and activation. Created with BioRender.com. NLRP3, NLR family pyrin domain containing 3; TLR4, toll-like receptor 4; NF-κB, nuclear factor-κB.

The NLRP3 inflammasome is a crucial cytoplasmic innate immune complex composed of the NLRP3 receptor, ASC adaptor protein, and pro-caspase-1. It serves as a core machinery driving exaggerated inflammation and pyroptosis following ischemia-reperfusion injury, and also accounts for the persistent non-healing state of chronic wounds (69). NLRP3 inflammasome activation follows a canonical two-step signaling paradigm. In the priming signal, DAMPs trigger TLR4/NF-κB activation, which upregulates the basal expression of NLRP3 and pro-IL-1β. The secondary activating signal is initiated by mitochondrial ROS accumulation and intracellular potassium efflux. Reduced cytoplasmic K^+^ concentration induces NLRP3 conformational rearrangement, exposing the pyrin domain (PYD). NLRP3 subsequently recruits ASC via homotypic PYD-PYD interactions, promoting the assembly of characteristic filamentous ASC specks. ASC further recruits pro-caspase-1 through the caspase activation and recruitment domain (CARD) homotypic interaction, termed CARD-CARD binding. Accumulated pro-caspase-1 undergoes autocatalytic cleavage to generate enzymatically active mature caspase-1. Activated caspase-1 mediates two pivotal pro-inflammatory events. First, it cleaves pro-IL-1β and pro-IL-18 to produce biologically active pro-inflammatory cytokines. Second, it cleaves gasdermin D (GSDMD), releasing the N-terminal fragment that oligomerizes on the plasma membrane to form functional pores. This process triggers cell pyroptosis and massive release of intracellular pro-inflammatory contents, further amplifying local aseptic inflammation (70, 71) (**Figure 4**).

Within the proposed model, the NLRP3 inflammasome functions as a key inflammatory signal amplifier. Strong TLR4/NF-κB signaling (the number of R) potentiates NLRP3 activation, further elevating inflammatory output and driving higher R values. In contrast, dominant STAT3 signaling (the denominator of R) suppresses NLRP3 activity, which facilitates R value reduction. Exosomes efficiently block this pro-inflammatory amplification loop by scavenging excessive ROS and directly inhibiting NLRP3 or caspase-1 activity (72). In flap IRI, exosome-mediated NLRP3 inhibition breaks the vicious cycle of “inflammation-pyroptosis-inflammation” and alleviates acute tissue damage (73). In chronic wounds, sustained NLRP3 suppression terminates persistent inflammatory arrest, permits transition from the inflammatory phase to the proliferative phase, promotes granulation tissue formation and re-epithelialization, and ultimately accelerates chronic wound healing (74).

### 4.6 MAPK signaling pathway: the rapid responder to microenvironment cues and regulator of threshold sensitivity of the model

**Figure 5:**
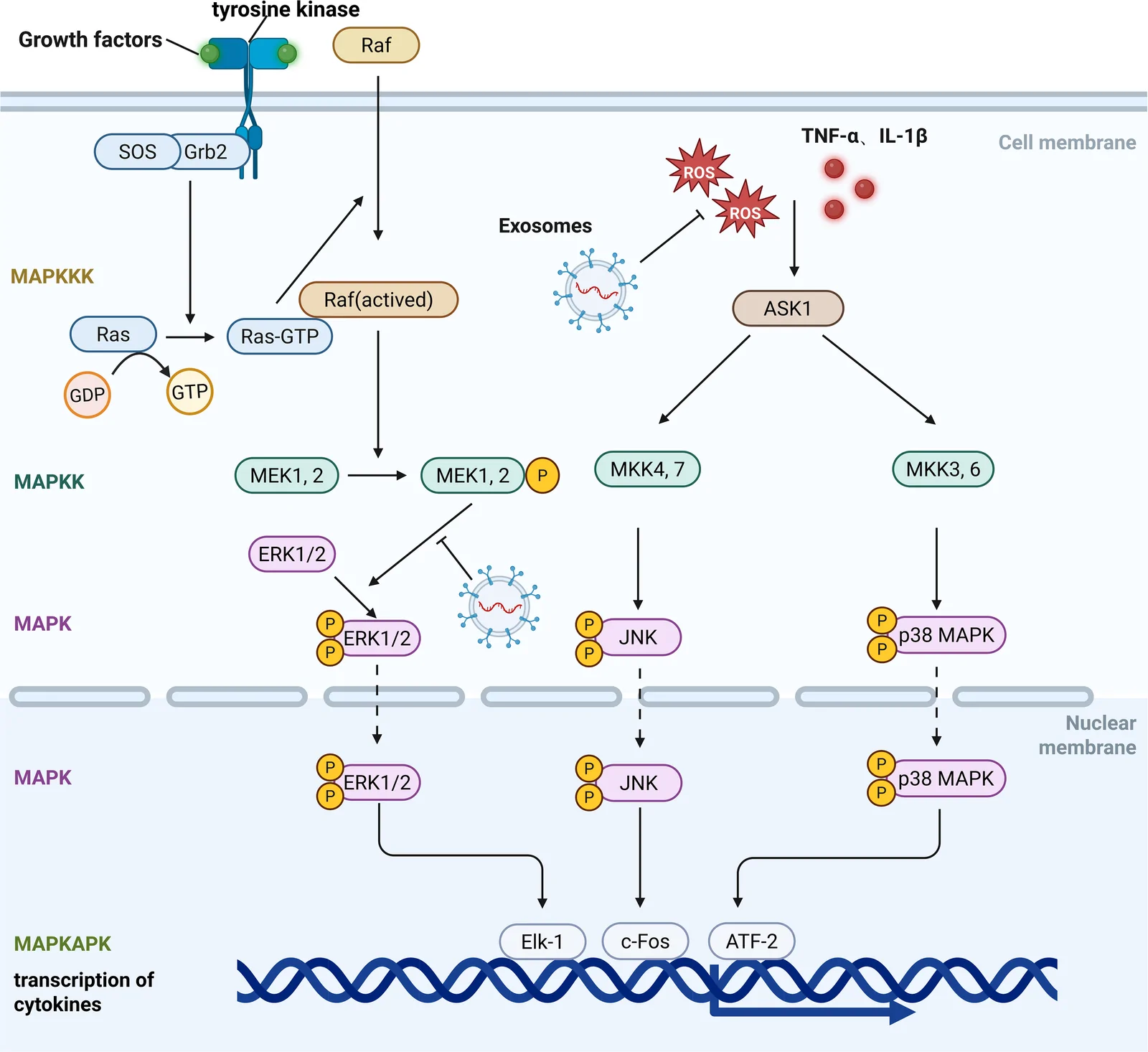
Mechanism diagram of exosomes regulating the MAPK signaling pathway. Stimuli including growth factors, inflammatory cytokines and ROS can activate three branches of MAPK cascades: ERK, JNK, and p38 MAPK. Activated MAPK proteins translocated into the nucleus to modulate transcription factors and mediate the expression of inflammation-associated target genes. Exosomes interfere with this activation process by targeting key nodes within the cascade. Created with BioRender.com. MAPK, mitogen-activated protein kinase; ROS, reactive oxygen species; ERK, extracellular signal-regulated kinase; JNK, c-Jun N-terminal kinase.

The MAPK family constitutes a core cascade that mediates inflammatory signal amplification, encompassing three well-characterized subfamilies: p38 MAPK, extracellular-regulated protein kinases 1/2 (ERK1/2), and c-Jun N-terminal kinase (JNK). MAPK signaling proceeds via a canonical three-tiered kinase cascade: MAPKKK phosphorylates and activates MAPKK, which further dual-phosphorylates downstream MAPK (75). In the ERK1/2 branch, growth factors bind to receptor tyrosine kinase and promote Ras-mediated GDP-for-GTP nucleotide exchange through the Grb2-SOS adaptor complex. GTP-bound activated Ras recruits Raf to the plasma membrane and triggers its activation. As the first-tier kinase MAPKKK, Raf phosphorylates serine residues on MEK. Activated MEK then dual-phosphorylates threonine and tyrosine residues within the TEY motif located in the ERK1/2 activation loop, driving ERK1/2 nuclear translocation to govern transcription of proliferation-associated genes (76). In the p38 MAPK and JNK branches, abundant ROS and pro-inflammatory cytokines (TNF-α, IL-1β) generated during flap IRI activate upstream apoptosis signal-regulating kinase 1 (ASK1). Subsequently, mitogen-activated protein kinase kinase 3/6 (MKK3/6) and MKK4/7 respectively phosphorylate the TGY motif of p38 and the TPY motif of JNK (77). Within chronic wounds, persistent phosphorylation of p38 and JNK locks macrophages in the pro-inflammatory M1 phenotype; consequently, wound healing stagnates in the inflammatory phase and fails to progress toward the proliferative phase (78) (**Figure 5**).

Within the proposed model, the MAPK pathway functions as a rapid responder to microenvironment cues, whose activation modulates the sensitivity of model threshold *R_th_*. For instance, sustained p38/JNK phosphorylation elevates *R_th_* and prevents exosomes from switching to pro-regenerative programs, which largely accounts for the poor healing ability of chronic wound. By restraining excessive p38/JNK activation, exosomes lower *R_th_*, enabling pro-regenerative programs to be initiated at relatively low R values (79). Meanwhile, moderate ERK activation cooperates with STAT3 to facilitate cellular proliferation. Therefore, MAPK does not serve as an independent decision-making module; rather, it dynamically tunes the overall magnitude of the R ratio.

Collectively, these analysis indicate that the five core pathways do not operate in isolation. Instead, they assemble an integrated decision-making network centered on the TLR4/NF-κB-STAT3 signal-intensity ratio R. By sensing microenvironment stimuli including inflammatory cytokines, ROS and hypoxia, exosomes dynamically remodel their cargo composition to fine-tune R values. This property underpins their dual therapeutic outputs: suppression of overwhelming acute inflammatory bursts in IRI, restraint of persistent low-grade inflammation, and promotion of tissue regeneration throughout the repair process. This model provides a unified interpretive framework for conflicting experimental observations regarding exosome-based wound immunotherapy and informs future rational design of engineered exosome therapeutics.

## 5 Engineering strategies and clinical translation roadmap based on the signal balance decision model

The proposed “TLR4/NF-κB-STAT3 signal balance decision model” indicates that the therapeutic performance of exosomes is determined by their capacity to modulate the TLR4/NF-κB to STAT3 signal ratio (R). Accordingly, the design of engineered exosomes should shift from static single-function enhancement to intelligent R-value modulation, enabling exosomes to autonomously switch between anti-inflammatory and pro-regenerative programs in response to dynamic wound microenvironmental conditions.

### 5.1 R-value-tailored engineering strategies

#### 5.1.1 Isolation and standardized manufacturing

Current exosome isolation techniques, including ultracentrifugation, size-exclusion chromatography, polymer precipitation, and microfluidics, possess distinct advantages and limitations (80–82). Clinical-grade production requires comprehensive optimization of purity, yield and GMP compliance. Notably, different isolation protocols reshape the functional molecular profile of exosomes, thereby altering their intrinsic baseline capacity for R-value regulation. Therefore, quality control (QC) should incorporate functional potency indicators in addition to routine characterization of particle size and canonical exosome markers (CD9, CD63, CD81). For instance, the phosphorylation ratio of p65 to STAT3 in standard macrophages models after exosome intervention can serve as a quantitative potency index to guarantee batch-to-batch consistency. This R-value-based potency assay establishes a four-layer quantitative QC system, enabling full-chain standardized supervision from exosome preparation to clinical release.

#### 5.1.2 Cargo engineering: presetting R-value functional bias

Parental cell preconditioning and direct cargo loading are two major strategies to preset exosomes with defined R-regulating bioactivity, namely R-reduction for anti-inflammation and R-elevation for pro-repair. For parental cell preconditioning, low-dose TNF-α or IL-1β stimulation enriches miR-146a in MSCs, potentiating the R-reducing capacity of derived exosomes (83). In contrast, IL-4/IL-13 preconditioning upregulates the expression of IL-10 and TGF-β, endowing exosomes with an R-elevating pro-regenerative bias (84). For direct cargo manipulation, functional miRNAs (e.g., NLRP3-inhibiting miR-223) or anti-inflammatory proteins (e.g., IL-10) can be encapsulated into exosomes via electroporation, ultrasonivation, or mechanical extrusion. Furthermore, genetic modification of parental cells to overexpress target molecules fused with exosomal sorting sequences achieves stable, controllable cargo packaging (85, 86).

#### 5.1.3 Surface modification and microenvironmental responsiveness

To equip exosomes with microenvironment-adaptive R-sensing capability, multiple engineering approaches can be adopted. Targeted surface decoration, such as displaying anti-CD86 or anti-CD206 nanobodies, enables exosome enrichment in pro-inflammatory M1 or reparative M2 macrophages, respectively (87). Stimuli-responsive polymeric coatings can be engineered to release R-reducing cargo rapidly in high-R hyperinflammatory microenvironments and sustain R-elevating cargo release in low-R repairing microenvironments. In addition, biomimetic hybridization with liposomes or cell membrane vesicles effectively prolongs the circulatory half-life exosomes (88).

#### 5.1.4 Delivery system synergy

Immobilizing exosomes within hydrogels, microneedles or electrospun scaffolds markedly prolongs local retention and improves bro-stability (89). Based on the model mechanism, rapidly degradable hydrogel with 24–72 hour release kinetics are optimal for acute flap IRI to match the transient hyperinflammatory storm window. In comparism, slowly degradable hydrogels with 7–14 day release kinetics are suitable for chronic wounds to sustain a low-R immune homeostasis. These two graded delivery strategies precisely accommodate stratified clinical populations characterized by high-R acute ischemic flaps and moderate-R chronic non-healing wounds. Future clinical trial design will adopt this stratified delivery paradigm, matching exosome-embedded scaffolds with tailored degradation cycles for subjects with distinct baseline R levels.

### 5.2 Core clinical translation barriers and model-oriented solutions

#### 5.2.1 Standardization and quality control

Exosomes derived from distinct cell sources display substantial batch-to-batch functional heterogeneity, while unified official quality release criteria are still absent (90). Based on the proposed R-ratio model, functional potency assays targeting R-value modulation should be incorporated into routine release standards, complementing the physicochemical tests required by the ISEV-MISEV2023 guidelines. This study establishes a standardized *in vitro* potency testing platform utilizing THP-1 macrophages differentiated with phorbol 12-myristate 13-acetate (PMA) as a universal reference cell line. Stimulation, administration and incubation procedures are fully standardized to quantify the protein expression of phosphorylated p65 (p-p65) and phosphorylated STAT3 (p-STAT3) after exosome intervention. The ratio of these two phosphorylation levels is calculated to generate dose-response potency curves, which quantify each batch’s capacity to modulate the TLR4/NF-κB and STAT3 cascades. This system enables functional calibration across different batches and addresses the evaluation limitation of purely physicochemical indicators (particle size, vesicle surface markers) that fail to reflect actual anti-inflammatory and pro-regenerative bioactivity.

#### 5.2.2 Long-term biosafety concerns

Allogeneic exosomes carry inherent risks of immunogenicity, tumorigenesis and fibrotic progression (91, 92). The model provides theoretical safety boundaries: excessive R reduction via full TLR4/NF-κB suppression may trigger immunosuppressive paralysis and elevate infection susceptibility, whereas persistent STAT3 hyperactivation caused by extreme R elevation induces aberrant tissue proliferation and pathological scarring. Therefore, dynamic monitoring of R values in perilesional tissue is mandatory during safety evaluation. We define the safe therapeutic window as sustained local R value ranging 0.5-2.0-fold relative to the physiological reference range of normal wound healing. Human umbilical cord-derived MSCs, characterized by low immunogenicity and negligible tumorigenic potential, are prioritized for production, and tumor-associated miR-21 levels are measured for every batch of exosomes.

#### 5.2.3 Unpredictable therapeutic efficacy and individualized administration

Multiple bottlenecks hinder consistent therapeutic outcomes: non-monotonic dose-response curves where excessive dosages suppress tissue repair (93); rapid exosome degradation within diabetic wound microenvironments rich in proteases; merely 0.1% systemic exosomes accumulate at target wound sites after intravenous delivery (94); and up to 50% bioactivity loss following lyophilization (95, 96). This model proposes targeted solutions as follows. First, baseline R values are estimated by measuring the TNF-α/IL-10 ratio in wound exudate collected from patients. Individuals with high baseline R values receive R-reducing exosomes, while patients with moderate or low baseline R values are treated with R-elevating exosomes to achieve personalized therapeutic regimens. Second, local delivery via exosome-loaded hydrogel scaffolds avoids systemic dilution and shields exosomes from proteolytic degradation within wound microenvironments (89). Third, optimized lyoprotectant formulations (e.g., trehalose, polyethylene glycol) are adopted to retain at least 70% of the original R-modulating potency of fresh exosomes after lyophilization, and this threshold is incorporated into mandatory post-stability testing standards.

#### 5.2.4 Uniform quantitative evaluation benchmarks

The clinical translation of engineered exosomes is hampered by the lack of comprehensive, executable unified quantitative evaluation criteria. Wide fluctuations in functional activity across batches consequently create inconsistent administration protocols for clinical trials and reduce trial operability (97). Most existing studies only conduct physicochemical characterization including particle size and particle concentration, without functional potency readouts reflecting actual biological effects. Even batches passing all physicochemical inspections exhibit divergent capacities to regulate target signaling pathways, confounding therapeutic efficacy assessments in clinical trials (98).

To overcome this limitation, this study proposes a four-tier progressive quantitative quality control and release framework integrated with the TLR4/NF-κB-STAT3 R-ratio model. At the physicochemical tier, all samples comply with MISEV2023 guidelines published by ISEV; nanoparticle tracking analysis, western blot and transmission electron microscopy are performed to quantify particle concentration, detect canonical vesicle markers and identify vesicle morphology for fundamental physicochemical release inspection (99). At the functional potency tier, each batch’s R-modulating capability is quantified using the standardized reference macrophage platform. At the stability tier, clear thresholds for retained R potency are defined under cryopreservation and lyophilization conditions. At the safety threshold tier, permissible fluctuation ranges of local R values post-treatment are restricted. Combined evaluation of physicochemical indicators, functional potency, stability parameters and safety thresholds greatly improves the operability of clinical trial protocols for engineered exosomes.

### 5.3 Model-guided design of future clinical trials

Current clinical investigations on exosome-mediated wound repair predominantly focus on macroscopic wound healing outcomes while neglecting longitudinal dynamic monitoring of local immune signaling. A standardized analytical framework linking molecular biomarkers to clinical healing indices remains lacking. Existing studies merely observe superficial wound phenotypes, for instance, identifying serum miR-21-5p as a wound prognostic biomarker (100) and verifying that macrophage-derived exosomes improve flap survival (101). However, these studies fail to systematically interpret the intrinsic mechanisms by which exosomes remodel immune imbalance during wound repair. Based on the proposed TLR4/NF-κB-STAT3 signal balance decision model, this study establishes a stratified, quantitative, and implementable framework for future clinical trials. Detailed evaluation indicators, sampling time points, and stratified grouping principles are summarized in **Table 1**. The corresponding subject screening criteria are further optimized according to R-value stratification logic, compensating for the systemic limitations of current clinical trial designs.

**Table 1:** Clinical trial design based on TLR4/NF-κB-STAT3 signal ratio R.

Endpoint classification	Detectable hard clinical endpoints	Corresponding R-related molecular markers	Sampling time points	Clinical significance
Primary efficacy endpoints	1. Wound healing rate (percentage reduction of wound area at Day 7, 14 and 28) 2. Necrotic flap area of high-risk flaps at postoperative Day 7	Tissue p-p65/p-STAT3 ratio (R value), TNF-α/IL-10 ratio in wound exudate	Baseline, Day 7, Day 14, Day 28	Directly verify the correlation between R value and wound repair prognosis
Secondary efficacy endpoints	1. Duration of inflammatory response (days for subsidence of purulent exudation and erythema) 2. Granulation tissue coverage rate 3. Microvessel density (pathological sections) 4. Scar hypertrophy score (Vancouver Scar Scale)	M1/M2 macrophage ratio, NLRP3 inflammasome activation level, Treg/Th17 ratio	Baseline, Day 14, Day 28, Day 90	Elaborate the intermediate effects of R ratio on immune microenvironment modulation
Safety endpoints	1. Incidence of local adverse reactions including infection, hypersensitivity and erythema 2. Systemic inflammatory cytokine storm, hepatic and renal dysfunction 3. Long-term risk of aberrant proliferation and scar carcinogenesis during follow-up	Persistently excessively low R (risk of infection caused by immunosuppression); persistently excessively high R (fibrosis risk driven by sustained STAT3 hyperactivation)	Whole follow-up period up to Day 180	Define the safe range of R value (0.5–2 times the physiological baseline level)

To avoid the bias caused by single-marker-based efficacy evaluation, the present framework defines two independent evaluation dimensions: definitive clinical endpoints and exploratory molecular biomarkers, with complete statistical analysis protocols predefined. Primary clinical endpoints include the complete wound healing rate, proportion of necrotic flap area, and duration of local inflammation, which are quantitatively assessed through standardized wound photography and clinical examination to determine overall therapeutic efficacy. In contrast, the tissue p-p65/p-STAT3 phosphorylation ratio and wound exudate TNF-α/IL-10 ratio serve only as secondary exploratory biomarkers to characterize exosome-mediated immune homeostasis regulation, rather than as standalone efficacy judgment indices. Statistically, Spearman correlation analysis is applied to quantify the association between dynamic R-value fluctuations and core clinical outcomes throughout follow-up periods. Multiple linear regression models are further utilized to validate whether baseline R values can predict patient therapeutic responses, filling the gap in the correlation analysis between molecular signatures and clinical phenotypes in this field.

Stratified randomization represents the core innovative feature of this model-guided clinical trial design. All enrolled patients are classified into two cohorts according to baseline R values calculated from wound exudate at admission: high-risk flap IRI patients with baseline R>2.0, and chronic refractory wound patients with baseline R ranging from 1.0 to 2.0. After stratification, participants within each cohort are randomly assigned to receive R-reducing exosomes, R-elevating exosomes, or placebo interventions. The inclusion criteria balance population homogeneity and clinical generalizability, enrolling participants aged 18–75 years with wound areas of 2–20 cm^2^ and no systemic glucocorticoid or immunosuppressant administration within one month prior to enrollment. All participants are required to provide written informed consent and complete scheduled sampling and follow-up procedures. Patients with severe wound infection, malignant tumors, uncontrolled autoimmune diseases, or severe organ dysfunction are excluded. Pregnant or lactating females, participants enrolled in other biotherapeutic clinical trials within six months, and those unable to complete sampling and follow-up are also excluded. This strict screening strategy minimizes confounding factors interfering with the correlation analysis between R-value dynamics and clinical efficacy.

## 6 Limitations of the proposed signal balance decision model

The TLR4/NF-κB-STAT3 signal balance decision model established in this review delivers quantitative references for the quality control of engineered exosomes and the design of wound-related clinical trials, yet multiple inherent limitations remain unaddressed. First, the R ratio defined to evaluate immune homeostasis is deduced merely from reported pathway trends in published literature. Precise quantification of the critical *R_th_* has not been accomplished via standardized *in vitro* THP-1 macrophage models or *in vivo* wound animal models. Replicable experimental data are also absent to quantify how ROS, hypoxic conditions and diverse immune cell subsets within wound microenvironments modulate *R_th_*. At present, the model remains a qualitative analytical framework, and a unified standardized quantitative detection workflow needs further development. Second, the published studies supporting this model exhibit prominent heterogeneity. Investigations adopt exosomes derived from various MSCs including bone marrow, adipose tissue and umbilical cord, while inconsistent protocols for exosome isolation and purification, administration dosages and wound modeling strategies lead to drastically distinct baseline R values across experimental systems, hindering cross-study comparative analysis. Meanwhile, existing clinical trials exploring exosome-based wound repair generally enroll small cohorts, and large-sample multicenter datasets are lacking to validate the clinical applicability of this model. Finally, the wound immune network features intricate crosstalk among multiple signaling cascades. This model centers exclusively on the TLR4/NF-κB and STAT3 core pathways, and the synergistic or antagonistic effects of auxiliary cascades including TGF-β/Smad, NLRP3 and MAPK only partially interpret basic biological phenomena. The comprehensive feedback regulatory network formed by multiple pathways under complex pathological conditions such as combined wound infection and pathological fibrosis has not been fully elucidated. To overcome the above deficiencies, future research can unify standardized *in vitro* and *in vivo* detection systems to calibrate *R_th_*, integrate multi-pathway indicators to optimize the model structure, and supplement clinical evidence through stratified clinical trials, so as to further improve the generalizability of the proposed model.

## 7 Summary and prospects

Impaired healing of acute flap IRI and chronic wounds originates from disrupted immune microenvironment homeostasis, yet the two conditions exhibit distinct inflammatory patterns. IRI is characterized by TLR4/NF-κB-driven acute inflammatory storms, while chronic wounds sustain persistent low-grade inflammation and arrested regenerative programs. With inherent biocompatibility and multi-target regulatory capacity, exosomes remodel wound immune microenvironments at multiple levels, emerging as a core research hotspot for precise regenerative wound medicine.

**Figure 6:**
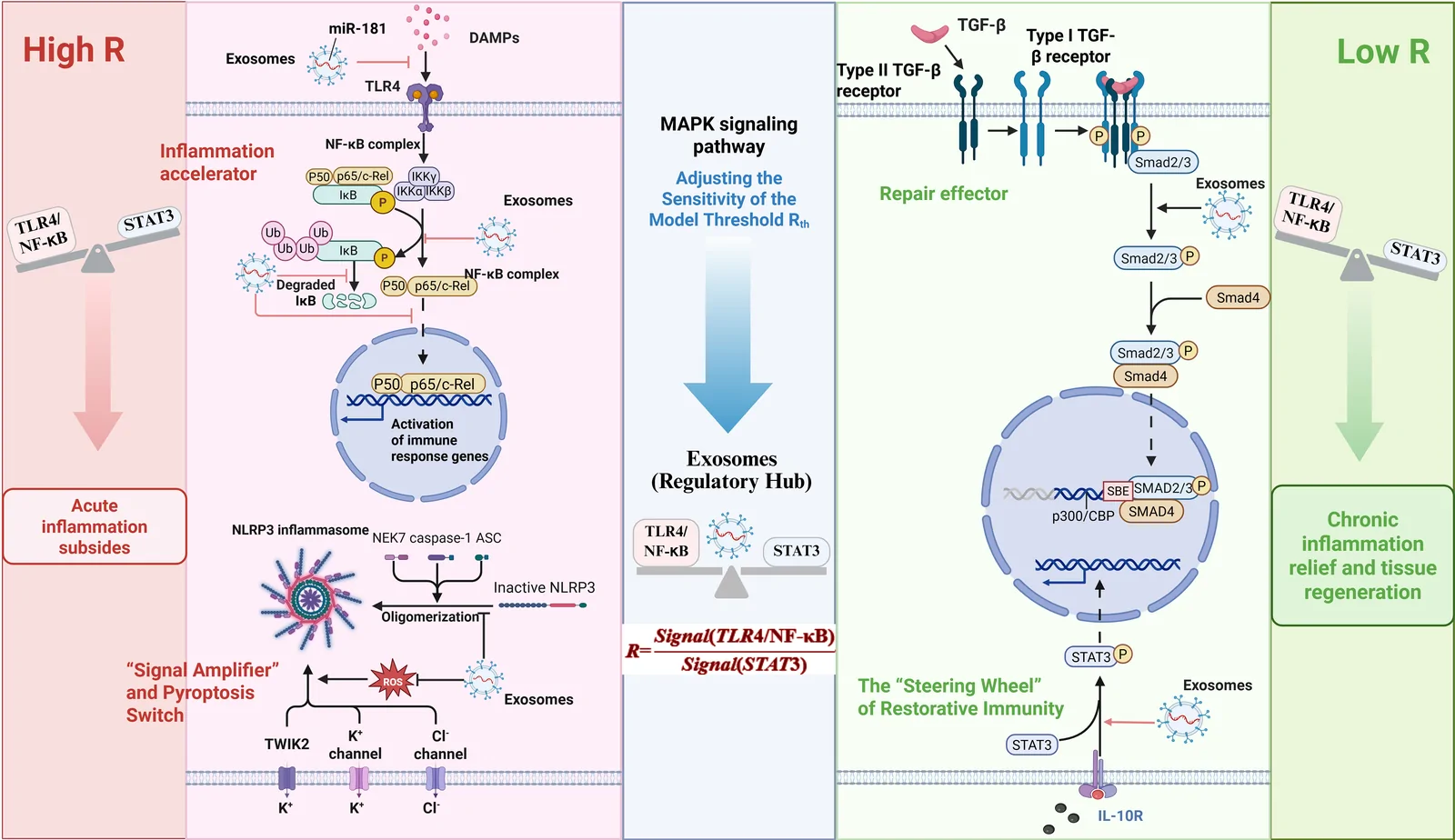
Exosome-mediated TLR4/NF-κB-STAT3 signal ratio (R-value) balance decision model. As core modulators of wound immune microenvironments, exosomes tune the R ratio of TLR4/NF-κB to STAT3 signaling. A high R value represents predominant TLR4/NF-κB activity, which initiates inflammatory cascades and triggers the NLRP3 inflammasome-pyroptosis axis to counteract acute inflammatory storms. The MAPK pathway controls the sensitivity of the critical threshold R_th_ in this model. When the R value declines, STAT3 signaling is preferentially activated, triggering reparative immunity through the TGF-β/Smad and IL-10/STAT3 axes to relieve sustained chronic inflammation and facilitate tissue regeneration. This model attributes the dual opposing functions of exosomes to dynamic shifts in the balance of these signaling pathways. Created with BioRender.com. TLR4, toll-like receptor 4; NF-κB, nuclear factor-κB; STAT3, signal transducer and activator of transcription 3; NLRP3, NLR family pyrin domain containing 3; MAPK, mitogen-activated protein kinase; TGF-β, transforming growth factor-β.

This review systematically dissects the divergent regulatory mechanisms of exosomes in acute and chronic wounds, identifying bidirectional modulation centered on the crosstalk between TLR4/NF-κB and STAT3 signaling. In hyperinflammatory IRI microenvironments, exosomes primarily suppress the TLR4/NF-κB inflammatory cascade to terminate acute tissue damage. In chronic wounds, they preferentially activate STAT3 signaling to restart tissue regeneration. Auxiliary regulatory networks consisting of TGF-β/Smad, NLRP3, and MAPK pathways jointly modulate macrophage polarization, neutrophil infiltration, dendritic cell function and adaptive immune balance, ultimately determining wound repair outcomes (**Figure 6**).

To reconcile contradictory published mechanistic findings, this review innovatively proposes the TLR4/NF-κB-STAT3 signal ratio (R-value) balance decision model. The seemingly conflicting dual functions of exosomes are unified as dynamic shifts in pathway signal ratios driven by local microenvironmental cues. Breaking the limitations of prior qualitative descriptions focusing on single pathways or single cell phenotypes, this model establishes the first interpretable, quantifiable theoretical framework guiding exosome engineering. It resolves long-standing mechanistic controversies in the field and delivers novel strategies for intelligent exosome design and individualized wound treatment.

Despite its capacity to interpret most existing experimental observations, the model carries several research limitations: the critical R threshold has not been quantitatively verified via standardized *in vitro* and *in vivo* assays; exosomes derived from diverse cell sources display prominent functional heterogeneity; multi-pathway crosstalk networks remain incompletely characterized; and large-scale clinical datasets supporting its translational performance are absent. Future research can be advanced from three dimensions: mechanistic optimization, engineered modification, and clinical standardization.

At the mechanistic level, further research is required to resolve the spatiotemporal delivery patterns of core functional cargo within exosomes, completing the full mechanistic cascade of “exosome delivery – pathway regulation – immune remodeling – wound repair”. Priority should be given to validating the critical R threshold and the molecular machinery governing dynamic pathway switching, and clarifying how microenvironmental stressors including ROS, hypoxia and immune cell infiltration modulate immune balance decision points, so as to improve the accuracy and generalizability of the model.

At the engineering translation level, the design of exosomes will shift from conventional static functional enhancement to microenvironment-adaptive intelligent modulation. Guided by the R-value balance model, multiple strategies including parental cell preconditioning, targeted surface modification, precise cargo loading and stimuli-responsive sustained release delivery systems can be applied to manufacture functionalized R-reducing anti-inflammatory or R-elevating pro-regenerative exosomes. These tailored exosomes match the divergent immune demands of acute IRI and chronic wounds, overcoming current technical bottlenecks such as unstable therapeutic efficacy and poor resistance to pathological microenvironments.

At the clinical standardization level, an integrated quality control system complying with MISEV2023 guidelines is urgently needed. R-modulating potency assays should be incorporated into batch release functional standards to compensate for the deficiencies of conventional physicochemical characterization. Stratified administration and individualized intervention can be implemented according to baseline immune status of patient wounds. Unified administration dosages, therapeutic windows and delivery protocols will facilitate multicenter, large-sample randomized controlled trials to systematically validate the safety and long-term regenerative benefits of intelligent exosome therapy.

In conclusion, exosome-mediated immune remodeling provides an innovative therapeutic strategy for refractory wound repair. The signal balance decision model constructed herein achieves theoretical upgrades across three dimensions: from descriptive phenotypic observation to quantitative mechanistic interpretation, from empirical preparation to precise quality control, and from generalized treatment to stratified individualized intervention. Continuous refinement of model mechanisms and breakthroughs in exosome engineering technology will render stimuli-responsive exosomes a new standardized biological agent for clinical prevention of flap necrosis and radical treatment of intractable chronic wounds.
